# Impaired Cortical Cytoarchitecture and Reduced Excitability of Deep-Layer Neurons in the Offspring of Diabetic Rats

**DOI:** 10.3389/fcell.2020.564561

**Published:** 2020-09-16

**Authors:** Rocío Valle-Bautista, Berenice Márquez-Valadez, América D. Fragoso-Cabrera, Guadalupe García-López, Néstor Fabián Díaz, Gabriel Herrera-López, Ernesto Griego, Emilio J. Galván, José-Antonio Arias-Montaño, Anayansi Molina-Hernández

**Affiliations:** ^1^Departamento de Fisiología, Biofísica y Neurociencias, Centro de Investigación y de Estudios Avanzados del Instituto Politécnico Nacional, Mexico City, Mexico; ^2^Laboratorio de Investigación en Células Troncales y Biología del Desarrollo, Departamento de Fisiología y Desarrollo Celular, Subdirección de Investigación Biomédica, Instituto Nacional de Perinatología Isidro Espinosa de los Reyes, Mexico City, Mexico; ^3^Departamento de Farmacobiología, Centro de Investigación y de Estudios Avanzados del Instituto Politécnico Nacional, Mexico City, Mexico

**Keywords:** maternal diabetes, cerebral cortex development, neocortical cytoarchitecture, neocortical function, primary motor cortex

## Abstract

Maternal diabetes has been related to low verbal task scores, impaired fine and gross motor skills, and poor performance in graphic and visuospatial tasks during childhood. The primary motor cortex is important for controlling motor functions, and embryos exposed to high glucose show changes in cell proliferation, migration, and differentiation during corticogenesis. However, the existing studies do not discriminate between embryos with or without neural tube defects, making it difficult to conclude whether the reported changes are related to neural tube defects or other anomalies. Furthermore, postnatal effects on central nervous system cytoarchitecture and function have been scarcely addressed. Through molecular, biochemical, morphological, and electrophysiological approaches, we provide evidence of impaired primary motor cerebral cortex lamination and neuronal function in pups from diabetic rats, showing an altered distribution of SATB2, FOXP2, and TBR1, impaired cell migration and polarity, and decreased excitability of deep-layer cortical neurons, suggesting abnormalities in cortico-cortical and extra-cortical innervation. Furthermore, phase-plot analysis of action potentials suggests changes in the activity of potassium channels. These results indicate that high-glucose insult during development promotes complex changes in migration, neurogenesis, cell polarity establishment, and dendritic arborization, which in turn lead to reduced excitability of deep-layer cortical neurons.

## Introduction

Impairment in language, learning, memory, motor coordination, perception and problem-solving, as well as autism and schizophrenia, have been related to pregnancy diabetes (Ornoy et al., 1998, 1999, 2001; Stenninger et al., 1998; Dionne et al., 2008; Bolaños et al., 2015; Márquez-Valadez et al., 2018).

Neurodevelopmental impairment affecting cognition in the offspring of diabetic mothers was first reported by Churchill et al. (1969). Thereafter, several studies have reported opposite effects on neurodevelopment in children from diabetic mothers. While some reports indicate no effect (Persson and Gentz, 1984; Nelson et al., 2000), others report alterations in neurocognitive and psychomotor performance during childhood (Rizzo et al., 1997; Ornoy et al., 2001; Fraser et al., 2012; Nomura et al., 2012). However, prospective, longitudinal, and retrospective cohort studies from different countries support the hypothesis that diabetes during gestation hinders neurodevelopment in early and late infancy.

Prospective studies in early infancy reported lower mental development index (MDI) in the Bayley Scale of Infant Development in 1-year-old Israelite (Hod et al., 1999) and United States children (Nelson et al., 2003). However, only the Israelite cohort showed impaired psychomotor function as evaluated by the Bayley Psychomotor Development Index (PDI). Rizzo et al. (1991) showed an inverse correlation between MDI index and maternal β-hydroxybutyrate in United States 2-years-old children and between the Stanford–Binet score and maternal β-hydroxybutyrate and free fatty acid levels in 3, 4, and 5-years-old children. Other studies have also reported altered intellectual and behavioral development in 4-year-old Japanese (prospective cohort) and United States (retrospective cohort) children, according to the Tanaka-Binet Intelligence Scale and Wechsler Preschool and Primary Scale of Intelligence for Children, respectively (Yamashita et al., 1996; Nomura et al., 2012). Furthermore, a case-control longitudinal study including two Canadian birth cohorts (singletons and twins) reported that children (18 months to 7 years) show lower performance in expressive language and recognition memory (6 months), and lower non-verbal IQ at 48 and 60 months (Dionne et al., 2008).

In older children, Rizzo et al. (1997) reported that inadequate maternal metabolic regulation affected the intellectual performance and neuropsychological functions of children of 7- to 11-years-old. Two years later, Ornoy et al. (1999) showed that Israelian children under 9-years born to diabetic women had lower scores in verbal tasks and fine and gross motor skills evaluated by the Bruininks–Oseretsky test of motor proficiency. Fraser et al. (2012) reported lower cognition parameters by the Wechsler Intelligence Scale in 8-year-old children of diabetic mothers in the United Kingdom, and Bolaños et al. (2015) found lower I.Q. values and poor performance in graphic and visuospatial tasks in Mexican children (7- to 9-years-old) born to diabetic women.

Therefore, it appears that diabetes during pregnancy affects the neurodevelopment of offspring in the short and long term. However, this issue is difficult to study in humans due to the presence of confounding variables and the neurological complexity of the altered functions that involve several areas of the central nervios system (CNS) such as the cerebral cortex (motor, sensory, visual, and/or auditory), hippocampus, basal ganglia, amygdala, thalamus, and cerebellum. Animal models allow studying the effect of hyperglycemia at different stages of prenatal and postnatal development in a more controlled way, and obtaining brain tissue for evaluation at the cellular, functional, and molecular levels.

Few studies have analyzed neocortical development in embryos without neural tube defects or the offspring of diabetic dams. Fu et al. (2006) studied the effect of high glucose (30 mM) on the proliferation and differentiation of cortical neural stem cells (NSCs) *in vitro* (embryo day 13, E13) and *in vivo* (E14), finding increased cell death, reduced cell proliferation, and increased neuronal differentiation. However, it is not clear if these phenomena exist in embryos without neural tube defects. We previously reported increased neuron differentiation and early neuron maturation during the cortical neurogenic peak (E14) in embryos without neural tube defects from diabetic rats, events that were associated to increased expression of neurogenic factors and the appearance of the high molecular mass isoform of the Microtubule-associated protein 2, MAP2 (Solís et al., 2017). The effect of high glucose on cerebral cortex development has been addressed. However, it is also important to elucidate the postnatal consequences.

In general, projection neurons originated during embryo corticogenesis are glutamatergic, and possess unique morphological, functional, and expression profiles (Shen et al., 2006; Molyneaux et al., 2007; Gaspard et al., 2008). Transcription factors have been employed as markers for projection neurons, namely Satb2 (layers II/III and IV), Tbr1 (layers V/VI), and FoxP2 (layers V/VI), due to their relevance in the formation of cortical pathways and functions. Satb2 regulates the fate of callosal projection neurons, layer VI cortico-thalamic neurons, and layer V subcortical projection neurons, through gene repression and activation (Alcamo et al., 2008; Britanova et al., 2008; McKenna et al., 2015). Accordingly, Satb2 mutations cause language and behavioral impairment, as well as intellectual deficit (Docker et al., 2014). Tbr1 participates in frontal identity and is essential for the specification and projection patterns of layer VI cortico-thalamic neurons (Hevner et al., 2002; Bedogni et al., 2010). Tbr1 knockout mice show corpus callosum agenesis indicating the participation of this protein in promoting the formation of callosal projections (Hevner et al., 2001). Foxp2 is a factor linked to language disorders with difficulties in coordinating and executing orofacial movements, structural aberrations in cortical and subcortical regions, and changes in gray matter (Vargha-Khadem et al., 1995, 1998; Lai et al., 2001; Watkins et al., 2002; Liegeois et al., 2003). Homozygous Foxp2 knockout mice show lethality at 3–4 weeks after birth, learning difficulties, alterations in motor development, and the absence or severe reduction in the frequency and duration of ultrasonic vocalizations, whereas heterozygous animals show reduced vocalization, with short and abnormal sounds (Shu et al., 2005; Fujita et al., 2008; Groszer et al., 2008; Gaub et al., 2010; Castellucci et al., 2016, 2017).

Our research group is interested in studying the effect of maternal diabetes on motor performance, given its importance for language execution and fine and gross movements. We, therefore, explored changes in the primary motor cortex (M1), the main source of cortical synaptic information to the basal ganglia, a group of neuronal nuclei critically involved in the planning, execution, and control of voluntary movement (Terumitsu et al., 2006; Enard, 2011; Simonyan and Horwitz, 2011).

We report here changes in the expression and distribution of SATB2, FOXP2, and TBR1 in M1 of neonates (Postnatal day 0; P0) and infants (P21) of diabetic rats, as well as aberrant distribution, impaired migration, and polarity establishment accompanied by electrophysiological changes, in deep cortical layer neurons in the P21 offspring of diabetic rats. These findings contribute to the knowledge of the postnatal consequences of *in utero* high glucose in the cortex and, therefore, to the understanding of the neurological motor alterations reported in humans in association with maternal diabetes.

## Materials and Methods

All experiments and animal manipulations were in accordance with the Animal Welfare Assurance (A5281-01), the ‘Guide for the Care and Use of Laboratory Animals’ (N.I.H. Publication No. 80-23, revised 1978) and ‘Norma Official Mexicana para la Producción, Cuidado y Uso de Animales de Laboratorio’ (NOM-062-ZOO-1999). The protocol was approved by the Research and Animal Care (CICUAL), Biosecurity, and Ethics committees of Instituto Nacional de Perinatología Isidro Espinosa de los Reyes (protocol 3230-21202-01-2015).

### Hyperglycemia Induction

Female Wistar rats (230–300 g) were maintained at Instituto Nacional de Perinatología Isidro Espinoza de los Reyes animal facilities under standard conditions (12:12 h light/dark cycle, 21 ± 2°C and, 40% relative humidity) with access to water and food *ad libitum*. A vaginal smear confirmed the presence of spermatozoids the morning after mating, and this time point was defined as E0.5. Thereafter, pregnant rats were housed individually.

At day 5 of pregnancy, rats were divided into two groups, control and diabetic, which received a single intraperitoneal injection of a citrate-buffered solution (vehicle; pH 6.4) or streptozotocin (STZ; 50 mg/kg body weight in 250 μl vehicle; Sigma Aldrich, St. Louis, MO, United States), respectively. After 24 h, plasma glucose levels were determined in a drop of blood obtained by puncture of the tail vein by using an electronic glucometer (ACCU-Check Performa, Roche Diagnostics, Basel, Switzerland). Rats injected with STZ and glucose levels higher than 200 mg/dl were assigned to the diabetic group, whereas animals with less of 200 mg/dl of glucose were discarded. Animals that received citrate-buffered solution with glucose levels 96–120 mg/dl were included in the control group.

For experiments involving embryos, pregnant rats were killed by decapitation at E14. The embryos were extracted, washed in cold phosphate-buffered saline (PBS, pH 7.4), and fixed by immersion in Boüin’s solution (15:15:1 saturated picric acid aqueous solution: 40% formalin:glacial acetic acid) followed by immersion in 15 and 30% sucrose gradients for 24 h each. After fixation, embryos were frozen in isopentane (Sigma-Aldrich) embedded in TissueTek (Sakura Finetek Europe, Flemingweg, Netherlands) and coronal slices (10 μm thick) were obtained with a cryostat (Leica CM1850 UV, Wetzlar, Germany).

For postnatal analyses, male neonates (P0) were weighted, glucose levels were measured (Supplementary Table 1), and total pups were counted (Supplementary Table 2). The neonatal tissue was processed within the first 2 h of birth. Fresh tissue was used for receptor binding and electrophysiological analysis (P21), while tissue fixed by transcardiac perfusion (P0 and P21) was used for immunofluorescence.

Pups (P0 and P21) were anesthetized with sodium pentobarbital (50 mg/kg body weight, i.p.; Laboratorios PiSA, Mexico City, Mexico), and transcardiac perfusion, through the ascending aorta, was performed with PBS (7.5 ml) followed by Boüin’s solution as fixator (7.5 ml), at room temperature and a constant flow rate of 0.5 ml/min (Masterflex L/S Digital Miniflex Pump, Vernon Hills, IL, United States). After decapitation, brains were removed and placed in Boüin’s solution for 72 h and then placed in 30% sucrose solution for 72 h for cryopreservation, embedded in TissueTek, frozen and maintained at −80°C until used.

### Quantitative Reverse Transcription-Polymerase Chain Reaction (qRT-PCR)

The frontal cerebral cortex from one pup per litter (*n* = 3–4) was dissected, and total RNA was obtained using TRIZOL reagent (Invitrogen, Carlsbad, CA, United States), following the procedure recommended by the manufacturer. First-strand cDNA was synthesized from 1 μg of total RNA in a mix containing 0.5 μg Oligo (dT), 1 mM dNTPs, 1 U RNAsin Ribonuclease Inhibitor and 5 U AMV Reverse Transcriptase (Promega, Madison, WI, United States).

The dynamic range was established for each amplification product to determine the fluorescence threshold and reaction efficacy. The presence of a single amplified product was ensured by a single peak in the melting curves. Preamplifier cDNA (15 cycles using the Gotaq D.N.A. polymerase; Promega) was used to evaluate changes in the relative expression of FoxP2, Tbr1, and Satb2 using the 2^–ΔΔCT^ method (Livak and Schmittgen, 2001). Gapdh amplification was used as the internal control.

qPCR was performed in a Rotor-Gene 6000 thermocycler (Qiagen, Germantown, MD, United States). The commercial KAPA^TM^ SYBER FAST^®^ qPCR mix (KAPA Biosystems, Wilmington, MA, United States), the corresponding cDNA, 20 pmol of the forward (F) and reverse (R) primers, in a total volume of 10 μl was used for qPCR reactions. The amplification conditions were: 95°C for 10 min followed by 40 cycles at 95°C for 30 s, 63°C (Foxp2), 60°C (Tbr1), 57°C (Satb2) and 58°C (Gapdh) for 15 s and 72°C for 15 s.

Primer sequences were as follows:

Foxp2, F: 5′-GAAAGCGCGAGACACATCG-3′, R: 5′-GAA GCCCCCGAACAACACA-3′;

Tbr1, F: 5′-GGAAGTGAATGAGGACGGCA-3′, R: 5′-TGG CGTAGTTGCTCACGAAT-3′;

Satb2, F: 5′-CCGCACACAGGGATTATTGTC-3′, R: 5′-TCC ACTTCAGGCAGGTTGAG-3′;

Gapdh F: 5′-GGACCTCATGGCCTACATGG-3′, R: 5′-CCC CTCCTGTTGTTATGGGG-3′.

### Immunofluorescence

One or two E14, P0 or P21 animals were used per litter from 3–5 different litters (*n* = 3–5), and telencephalic (E14) or cortical (P0 and P21) coronal slices (10 μm thick) were obtained using a cryostat (Leica CM1850 UV) and recovered in glass slides pre-treated with Poly-L-lysine (Sigma-Aldrich, St. Louis, MO, United States).

Brain slices were washed with PBS, blocked and permeabilized (10% normal goat serum, and 0.3% Triton-X100 in PBS) during 1 h. Autofluorescence was blocked with TrueBlack lipofuscin autofluorescence quencher (Biotium, Hayward, CA, United States), and slices were then incubated overnight at 4°C with the following primary antibodies: rabbit polyclonal anti-SATB2 (1:250, RRID:AB_2301417; Abcam, Cambridge, MA, United States), anti-TBR1 (1:100, RRID:AB_2200219; Abcam), anti-FOXP2 (1:250, RRID:AB_2107107; Abcam), and anti-MAP2 (1:500; RRID:AB_369978; GeneTex, Irvine, CA, United States), or mouse monoclonal anti-Reelin (1:250, RRID:AB_2285132; Millipore, Burlington, MA, United States) and anti-Nestin (1:100, RRID:AB_11175711; GeneTex). The day after, slices were washed with PBS, and incubated for 1 h with the following fluorescent secondary antibodies: Alexa Fluor 488 anti-rabbit IgG (1:1000, RRID:AB_143165; Thermo Fisher Scientific, Waltham, MA, United States) and Alexa Fluor 568 anti-mouse IgG (1:1000, RRID:AB_2534072; Thermo Fisher Scientific). Nuclei were counterstained with DAPI (5 ng/ml; Sigma-Aldrich). Negative controls were performed by omitting the primary antibodies (Supplementary Figure 1).

Micrographs of the frontal cortex (E14) and M1 (P0 and P21) were obtained using an epifluorescence microscope (Olympus IX81, Shinjuku, Tokyo, Japan) with a charge-coupled device camera (ORCA-FLASH 2.8 CCD model C11440; Hamamatsu, Honshu, Japan) and the images were processed with Adobe Photoshop CS6 (San Jose, CA, United States).

For fluorescence quantification, the exposition and gain values used for the first micrograph from a control sample for each marker were maintained for all subsequent acquisitions, and the fluorescence was measured using Fiji software (Schindelin et al., 2012). The results were expressed as a percentage of the fluorescence obtained in the control samples.

### Western Blot

Total protein was obtained from the fresh frontal cerebral cortex of one pup from five different litters (*n* = 5 experiments) per group. The tissue was homogenized in lysis buffer (25 mM Tris-HCl, pH 7.4, 1% IGEPAL, 100 mM NaCl) containing a protease inhibitor cocktail (Amresco, Solon, OH, United States) and centrifuged (13,000 × *g*, 4°C, 10 min). The supernatant was recovered, the amount of protein was determined by the Bradford method (Bradford, 1976), and aliquots were maintained at −80°C until use.

Electrophoresis of protein samples (20 μg for SATB2 and TBR2, or 80 μg for FOXP2) and 2.5 μl of a commercial molecular weight standard (1610376, Precision Plus Protein^TM^ WesternC^TM^ Blotting Standards, Bio-Rad or GTX50875, Trident Prestained Protein Ladder, GeneTex) was performed in denaturalizing 10% polyacrylamide gels with the Mini-Protean II system (Bio-Rad, Hercules, CA, United States). Proteins were transferred to nitrocellulose membranes (Amersham^TM^ Hybond TM-ELC, Buckinghamshire, United Kingdom) using the *Trans*-Blot^®^ semi-dry transfer cell system (Bio-Rad), as previously described (Villanueva, 2008). Membranes then were incubated overnight at 4°C with rabbit primary antibodies: anti-SATB2 (1:1500; Abcam), anti-TBR1 (1:500; Abcam), anti-FOXP2 (1:100; Abcam), and anti-GAPDH or anti-α-Actin (as internal controls; 1:1500, RRID:AB_1080976 or AB_370675; GenTex; AB_11174335). Secondary infra-red antibodies were IRDye 800CW and IRDye 680RD (1:10,000, RRID:AB_621848 and RRID:AB_2814912; LI-COR Biosciences, Lincoln, NE, United States). Blots were scanned in the Odyssey CLx system and analyzed with the Image Studio software version 4.0 (LI-COR Biosciences). The fluorescence for each protein was normalized to that obtained for Gapdh and expressed as a percentage of control values.

### Dendritic Orientation, and Sholl and Complexity Index Analyses

To evaluate dendritic orientation and to perform Sholl and dendritic complexity index (DCI) analyses, coronal brain slices from P21 pups from control and diabetic dams were analyzed with the FD Rapid GolgiStain^TM^ Kit (FD Neurotechnologies, Columbia, MD, United States) following the manufacturer instructions. Animals were decapitated, the brain was rapidly removed, and coronal sections (60 μm thick) were obtained with a cryostat. The slices were placed in the developer solution for 1 min and then washed in distilled water for 5 min, dehydrated in alcohol (50, 75, 96, and 100%; 5 min each), transferred to xylol and mounted with Entellan (Merck, Darmstadt, Germany).

Reconstructions of several micrographs (20×) in different planes following the pyramidal neuron arborization were made, scale printed, and the drawings obtained from the complete neurons were scanned for analysis. At least six neurons from the M1 per pair of layers (I–II, III–IV, and V–VI) of three pups from three different litters per group were analyzed.

To evaluate dendritic orientation, the angle of insertion of the apical dendrite to the cell body was quantified using Fiji software (Hashimoto-Torii et al., 2008; Schindelin et al., 2012). A dendritic orientation angle between 75 and 105° was considered normal, while angles >105° or <75° were considered abnormal (Hashimoto-Torii et al., 2008).

To evaluate the morphological characteristics of the dendritic arbor of the pyramidal neurons, the Sholl intersection profile was performed, by counting the intersections of the dendritic arbor with an imaginary circle of a given radius (10 μm increases), as a function of the distance from the soma (Sholl, 1953).

Based on the Sholl analysis, the dendritic complexity index (DCI), was calculated with the equation (Chameau et al., 2009):

$$DCI=\frac{\sum \mathrm{Ordinal}\mathrm{value}\mathrm{of}\mathrm{the}\mathrm{dendrite}+\#\mathrm{of}\mathrm{total}\mathrm{dendrites}}{\#\mathrm{of}\mathrm{primary}\mathrm{dendrites}}\times \mathrm{dendritic}\mathrm{arbor}\mathrm{length}$$

The ordinal value of a dendrite refers to the order in which a branch appears in relation to the first branch or primary dendrite (Chameau et al., 2009).

### Brain Slices

Three P21 pups from three different dams (*n* = 3) from each group were deeply anesthetized with an intraperitoneal injection of sodium pentobarbital (50 mg/kg), decapitated, and the brain was rapidly removed and placed in an ice-cold sucrose solution [in mM: 210 sucrose, 2.8 KCl, 2 MgSO_4_, 1.25 Na_2_HPO_4_, 26 NaHCO_3_, 10 D-(+)-glucose, 1 MgCl_2_, 1 CaCl_2_; pH 7.35 after saturation with 95% O_2_, 5% CO_2_].

The hemispheres were separated along the middle sagittal line, and the forebrain was cut and fixed to a vibratome (VT1000S; Leica, Nussloch, Germany) to obtain coronal slices (385 μm thick) containing the M1. The slices were incubated at 34°C for 30 min in a solution containing 125 mM NaCl, 2.5 mM KCl, 1.25 mM Na_2_HPO_4_, 26 mM NaHCO_3_, 4 mM MgCl_2_, 10 mM D-(+)-glucose and 1 mM CaCl_2_, saturated with 95% O_2_ and 5% CO_2_ for 30 min at 34°C, followed by at least 60 min at room temperature.

The slices were then placed in a perfusion chamber and perfused (2–3 ml/min) with artificial cerebrospinal fluid at 32–33°C [composition in mM: 125 NaCl, 2.5 KCl, 1.25 Na_2_HPO_4_, 26 NaHCO_3_, 2 MgCl_2_, 10 D-(+)-glucose and 2 CaCl_2_; pH 7.4 after saturation with 95% O_2_ and 5% CO_2_]. Neurons were visualized with a Nikon FN-S2N microscope coupled to an infra-red contrast camera (Nikon, Tokyo, Japan) and an immersion objective 40×. Borosilicate pipettes with a resistance of 5–8 MΩ were obtained with a horizontal puller (Flaming-Brown P-97; Sutter Instrument, Novato, CA, United States) and filled with an intracellular solution containing 135 mM *K*-Gluconate, 10 mM KCl, 1 mM EGTA, 10 mM HEPES, 2 mM Mg-ATP, 0.4 mM Na_2_-GTP, 10 mM phosphocreatine (pH 7.2–7.3 and 290–300 mOsm/l).

### Electrophysiological Recordings

Somatic recordings of pyramidal neurons from layers V and Vl (50–150 μm depth from the surface) were recorded in the whole-cell configuration of the patch clamp technique. Voltage- and current-clamp recordings were obtained with an Axoclamp 200B amplifier (Molecular Devices, Palo Alto, CA, United States), sampled and digitized at 10 kHz and filtered at 5 kHz (Digidata 1440A; Molecular Devices). Signals were acquired and analyzed off-line with pCLAMP 11.1 software (Molecular Devices). Additional filtering (50–60 Hz) was performed with a Hum Bug Electrical Noise eliminator (Quest Scientific, North Vancouver, BC, Canada). The resting membrane potential (RMP) was measured after initial break-in from gigaseal to whole-cell configuration. Pyramidal neurons were considered for analysis only if the access resistance was 5–9 MΩ and did not change 15% from the original value. Following the stabilization period (∼5 min), the cells were switched to the current-clamp mode, and different protocols were applied to determine input resistance (Rin), membrane time constant (τ), and the current required to elicit an action potential (rheobase) at the RMP of each recorded cell.

Rin was computed from the slope value obtained by plotting the voltage response to current injections of −30, 0, and +30 pA. The τ value was determined by adjusting an exponential function to the capacitive response elicited by current injection (30 pA, averaged from 10 consecutive sweeps). The I–V curves were constructed by applying current steps from −300 pA (30 pA steps, 1 s duration) until an action potential was elicited. Finally, the sag conductance or voltage drop was determined in response to a hyperpolarizing current step of -300 pA. The rheobase value and the action potential frequency were determined by applying current pulses from 0 to 500 pA (50 pA steps 1 s duration). The passive and active electrophysiological membrane properties were analyzed under basal or stimulated (histamine 1–100 μM) conditions, and analyses were strictly performed after the stabilization period.

Histamine acts as neuromodulator and neurotransmitter that, among several other functions, participates in spontaneous locomotion. The cerebral cortex is densely innervated by histaminergic fibers and expresses high levels of H_1_ receptors (H_1_Rs) (Inagaki et al., 1988; Inoue et al., 1996; Tagawa et al., 2001), and H_1_R activation results in neuron depolarization (McCormick and Williamson, 1991; Reiner and Kamondi, 1994; Wenger Combremont et al., 2016; Cilz and Lei, 2017; Wu et al., 2019).

Phase plots were constructed by plotting the time derivative of the somatic membrane potential (dV/dt) versus the somatic membrane potential of the first and fifth action potentials, and the mean ± the associated standard error (SEM) of the maximum rate values of the depolarization (Max DP dV/dt) or repolarization (Max RP dV/dt) phases of the action potential were plotted for the control and diabetic groups under basal and stimulated conditions.

### Radioligand Binding Assay

The frontal cerebral cortex of one P21 offspring from 5 different litters (5 experiments) per group was dissected, and the tissue was homogenized in a hypotonic solution (10 mM Tris-HCl, 1 mM EGTA, pH 7.4) and centrifuged (20,000 × *g* at 4°C for 20 min). The resulting pellet (total membranes) was re-suspended in 450 μl incubation buffer (50 mM Tris-HCl, pH 7.4).

H_1_R density was determined in membrane aliquots incubated for 60 min at 30°C in 100 μl incubation buffer containing 10 nM of the selective H_1_R antagonist [^3^H]-mepyramine (PerkinElmer, Waltham, MA, United States) in the presence or absence of 10 μM unlabeled mepyramine to determine non-specific and total binding, respectively. The incubation was terminated by filtration through Whatman GF/B glass microfiber filters (GE Healthcare Life Science, Marlborough, MA, United States) soaked in 0.3% polyethyleneimine for 2 h. The radioactivity retained in the filters was determined by scintillation counting (Soria-Jasso et al., 1997). Specific binding was determined by subtracting non-specific from total binding. Values were normalized to the amount of protein per sample (∼60 μg) determined by the bicinchoninic acid assay (Smith et al., 1985).

### Enzyme-Linked Immunosorbent Assay (ELISA)

Histamine content in the frontal cerebral cortex was determined by ELISA (cat. 17-HISRT-E01-RES. sensitivity 0.2 ng/ml, 100% histamine specificity), following the protocol recommended by the supplier (ALPCO^®^ immunoassays, Salem, NH, United States).

The cerebral cortex from one offspring from different litters (*n* = 3–5) per group was homogenized in 100 μl ice-cold PBS (Polytron PT 2100 Homogenizer, Kinematica, Luzern, Switzerland) and centrifuged (10,500 × *g* for 5 min). The supernatant was recovered and used to determine the histamine content using a reference curve constructed with 0, 0.5, 1.5, 5, 15, and 50 ng/ml histamine with duplicates by determining the absorbance at 450 nm in a multiple detection system (Glomax, Promega, Madison, WI, United States). Values were corrected by protein content determined by the Bradford method (Bradford, 1976).

### Statistical Analysis

Data are presented as means ± the associated standard error (SEM). Unpaired Student’s *t*-test was used for evaluating changes between groups after data passed the Shapiro–Wilk normality test. *F* test values obtained from the *t*-test are shown to validate that the two samples have similar standard deviation and thus similar variance. For comparison of the passive electrophysiological properties, repeated-measures two-way ANOVA and the *post hoc* Sidak’s multiple comparison test were used for differences in the same group, and repeated-measures two-way ANOVA and the *post hoc* Dunnett’s multiple comparison test to compare data in the absence (basal) or presence of histamine. *P*-values < 0.05 were considered significant. GraphPad Prism 6.0 software (GraphPad Sofware, La Jolla, CA, United States) was used for statistical analyses and graph construction.

## Results

### Altered Distribution and Expression of Cortical Layer Markers in the Offspring of Diabetic Rats

We first analyzed by immunofluorescence the laminar distribution of markers for superficial (SATB2) and deep (FOXP2 and TBR1) cortical layers in the M1 of neonates (P0) and P21 rats from control and diabetic dams.

**FIGURE 1:**
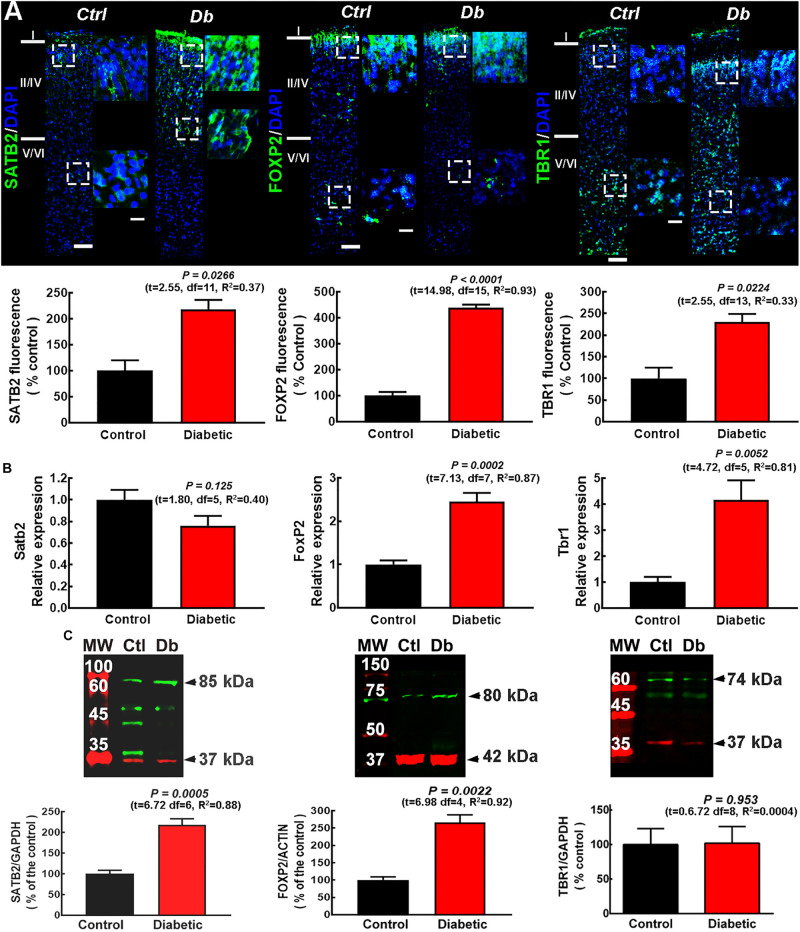
SATB2, FOXP2, and TBR1 expression and distribution in the neonatal primary motor cortex from control and diabetic dams. **(A)** Upper, representative micrographs (10×) of SATB2, FOXP2, and TBR1 (green) immunodetection and DAPI-stained nuclei (blue), at postnatal day zero (P0) from Control (Ctrl) and Diabetic (Db) groups (*n* = 3–4). Amplifications (400%) of the dotted squares from superficial and deep neocortex are shown on the right. Scale bars are 50 and 25 μm for 10× and zoom, respectively. Lower, quantitative analysis of the immunofluorescence of SATB2, FOXP2, and TBR1. Values are expressed as a percentage of the fluorescence of the controls and are means ± SEM from 3 experiments. The statistical analysis was performed with Student’s *t*-test. *F* test values (F, DFn, and Dfd) were: SATB2 (1.07, 5, and 6), FOXP2 (2.43, 10, and 5), and TBR1 (10.55, 2, and 3). **(B)** Quantitative analysis of qRT-PCR of Satb2, FoxP2, and Tbr1. Values were normalized to the relative expression of the control using the 2^–ΔΔ*CT*^ method, and are means ± SEM from 3–4 determinations. The statistical analysis was performed with Student’s *t*-test. *F* test values (F, DFn, and Dfd) were: SATB2 (1.33, 3, and 2), FOXP2 (3.95, 3, and 4), and TBR1 (1.63, 6, and 7). **(C)** Upper, representative Western blots for SATB2 (green, ∼85 kDa), FOXP2 (green, ∼80 kDA), TBR1 (green, ∼74 kDa), and the internal controls GAPDH (red, ∼37 kDa) or β-actin (red, ∼42 kDa). Bands on the left lane are the molecular weight ladders (M.W.). Lower, quantitative fluorometry analysis for SATB2, FOXP2, and TBR1. Values are expressed as a percentage of the fluorescence ratio of controls and are means ± SEM from 5 experiments. The statistical analysis was performed with Student’s *t*-test. *F* test values (F, DFn, and Dfd) were: SATB2 (3.04, 3, and 3), FOXP2 (5.97, 2, and 2), and TBR1 (1.08, 3, and 3).

As expected, at P0 for both the control and diabetic groups the layer distribution of these transcription factors in M1 was different from that reported for the adult cortex as well as from other cortical areas of P0 rats (Huang et al., 2013; Srivatsa et al., 2014). A significant increase in SATB2, FOXP2, and TBR1 immunofluorescence (2.18 ± 0. 17-, 4.38 ± 0. 12-, and 2.30 ± 0.18-fold, respectively) was observed in the neonatal M1 from the offspring of diabetic rats compared with control animals, (Figure 1A). An unexpected perinuclear/cytoplasmic mark for SATB2 and FOXP2 was observed in neonates (2 h after birth) in addition to the reported nuclear localization (Supplementary Figure 1).

The analysis by qRT-PCR of the expression of the transcription factors showed a significant increase in Foxp2 and Tbr1 mRNAs in the diabetic group compared with the control group (2.45 ± 0.20- and 4.17 ± 0.75-fold of control values, respectively), with no significant change for Satb2 expression (0.76 ± 0.09 of control values; Figure 1B). Western blot analysis showed a higher protein level for SATB2 (2.17 ± 0.15-fold) and FOXP2 (2.66 ± 0.22-fold) compared with the control group, with no change in TBR1 (1.02 ± 0.24-fold; Figure 1C).

**FIGURE 2:**
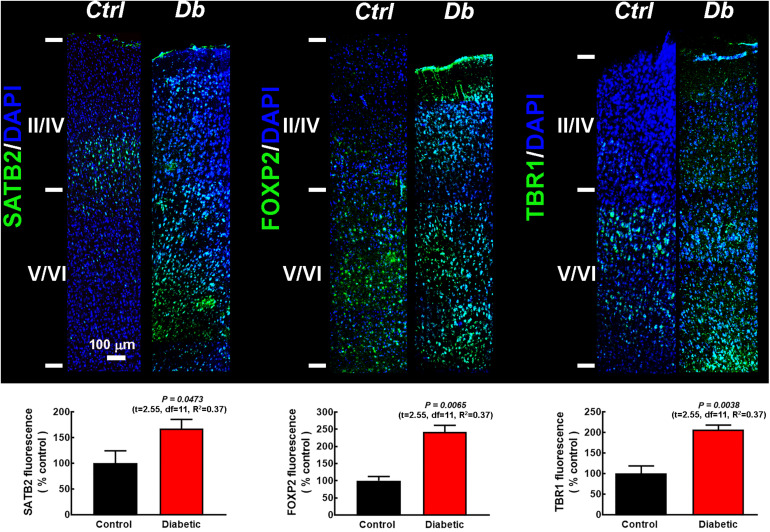
SATB2, FOXP2, and TBR1 distribution in the primary motor cortex of 21-days-old offspring from control and diabetic rats. **Upper**, representative micrographs (10×) of SATB2, FOXP2, and TBR1 (green) immunodetection and DAPI-stained nuclei (blue), at postnatal day 21 (P21) in the primary motor cortex from control (Ctrl) and Diabetic (Db) groups. Images are representative of 3–4 determinations. Scale bar = 100 μm. **Lower**, quantitative fluorometric analysis for SATB2, FOXP2, and TBR1. Values are expressed as a percentage of the fluorescence obtained in the controls and are means ± SEM from 3 experiments. *P*-values were obtained by unpaired Student’s *t*-test. *F* test values (F, DFn, and Dfd) were: SATB2 (1.10, 9, and 4), FOXP2 (1.73, 4, and 6), and TBR1 (2.68, 6, and 6).

To explore the distribution of the transcription factors at P21, we performed immunofluorescence assays in pups from diabetic and control dams. As expected for the motor cortex, deep layers were thicker than in other cortical areas, as evaluated by the immunoreactivity to the deep cortical layer markers FOXP2 and TBR1 in the control group (Figure 2). In pups from diabetic rats, an evident change in the immunostaining distribution was observed, with FOXP2 and TBR1 immunoreactivity extending to the superficial layers and SATB2 immunoreactivity out of its domain, extending to upper and lower cortical layers (Figure 2). Furthermore, at this stage of postnatal development, all the markers studied were higher in the diabetic group than the control group (Figure 2). As expected, at P21 all transcription factors studied showed a nuclear mark (Supplementary Figure 2).

#### Offspring From Diabetic Rats Shows Aberrant Migration and Dendritic Arborization

**FIGURE 3:**
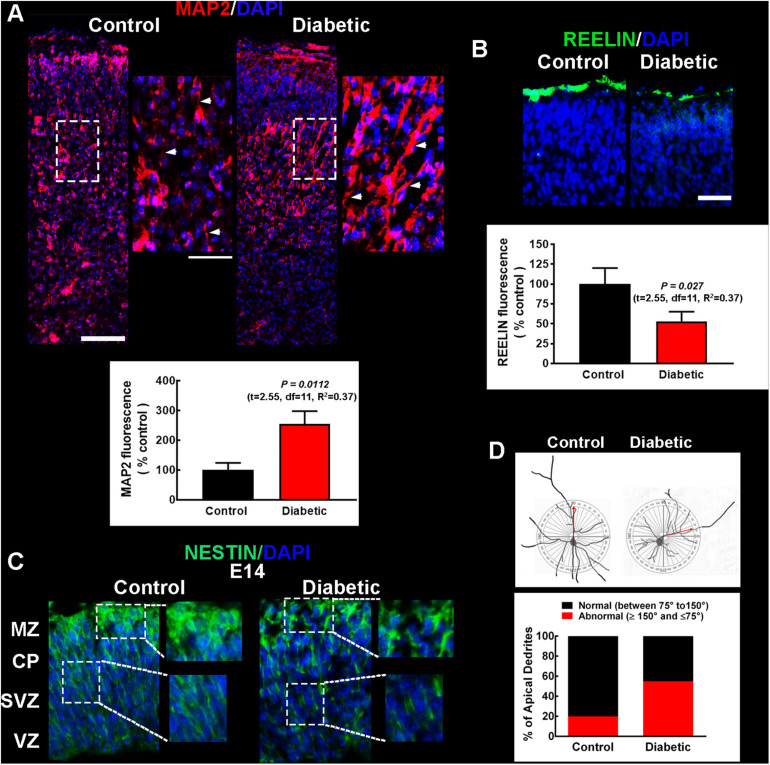
Migration and cell polarity markers in pups and embryos from control and diabetic rats. **(A)** Upper, representative micrographs (10×) of MAP2 immunodetection in the apical dendrites (red) of projection neurons of neonatal (P0) primary motor cortex from the offspring of control (left) and diabetic (right) rats. Electronic zooms (200%) of the dotted squares are shown on the right. White arrowheads indicate MAP2^+^ dendrites near DAPI-stained nuclei (blue), highlighting the atypical dendrite insertion into the soma in the diabetic group. Scale bars correspond to 100 and 50 μm for 10× and electronic zoom, respectively. Lower, quantitative fluorometric analysis for MAP2. Values are expressed as a percentage of the fluorescence obtained in the controls and are means ± SEM from 3 experiments. **(B)** Upper, representative micrographs (20×) of the Reelin^+^ mark (green) located in the upper cortical layers in P0 offspring from diabetic rats. Scale bar = 50 μm. Lower, quantitative fluorometric analysis for Reelin. Values are expressed as a percentage of the fluorescence obtained in the controls and are means ± SEM from 3 experiments. *P*-values were obtained in A and B by Student’s *t*-test. *F* test values (F, DFn, and Dfd) were: MAP2 (4.75, 7, and 5) and Reelin (1.85, 5, and 8). **(C)** Representative micrographs (20×) of the immunodetection of Nestin (green) radial glial scaffold in E14 embryos from control (left) and diabetic (right) rats. Electronic zooms (200%) from dotted areas are shown on the right, highlighting the impaired Nestin signal in embryos from the diabetic group. CP, cortical plate; MZ, marginal zone; SVZ, subventricular zone; VZ, ventricular zone. For all images, nuclei are observed in blue (DAPI). Images are representative of 3–4 determinations. **(D)** Upper, representative reconstructions of Golgi-Cox stained neurons from 21-days-old pups from control and diabetic dams, with a diagram of a cycle (180°) as a reference to determine the angle of insertion of the apical dendrite into the soma. Lower, quantitative analysis of neurons with normal or abnormal angle of insertion of the apical dendrite into the soma of the projection neuron. Values are expressed as a percentage and correspond to 27–30 neurons from different layers.

Changes observed in the distribution of the cortical markers suggested impaired cell differentiation and migration. Hence, apical dendrites were visualized at P0 by MAP2 immunofluorescence. The immunoreactivity showed increased MAP2 expression and angled dendrites (Figure 3A), suggesting impaired migration and dendritic growth in the offspring of diabetic rats.

To evaluate impaired migration and dendritic growth, Reelin immunofluorescence was performed in neonates, along Nestin staining to detect RG scaffolding for cortical neuron migration. In neonates from diabetic rats, the Reelin signal significantly decreased to 53 ± 12% of that observed in the control group (Figure 3B). Furthermore, in the dorsal telencephalon of E14 embryos from diabetic rats, the Nestin mark revealed a discontinuous pattern suggesting inadequate scaffold formation (Figure 3C), supporting the hypothesis of aberrant migration.

To corroborate impaired neuron migration, the angle of insertion of the apical dendrite into the cell body of pyramidal neurons was evaluated in Golgi-Cox stained neurons from M1 of P21 pups. A large fraction of these neurons had an inadequate angle of insertion in the diabetic group (55%, 2.80-fold of control value; Figure 3D) compared with control animals where the majority (80%) of the neurons presented an adequate angle.

**FIGURE 4:**
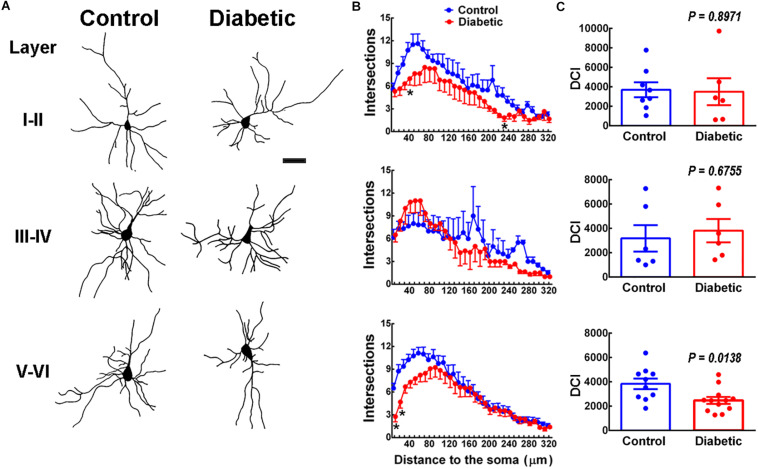
Dendritic arbor analysis in 21-days-old offspring from control and diabetic rats. **(A)** Representative reconstruction of Golgi-Cox stained projection neurons in cortical layers I to VI. Scale bar = 100 μm. **(B)** Sholl analysis. Values are means ± SEM from the number of intersections of a dendrite with respect to the distance from the soma in control (blue dots) and diabetic (red dots) groups. *P < 0.5, **P < 0.001 versus control values; multiple *t-*test. **(C)** Analysis of the dendritic complexity index (DIC) of neurons obtained from the offspring of control (blue bars) and diabetic (red bars) rats (*n* = 6–12 neurons per pair of layers). *P*-values were obtained by Student’s *t*-test with *t*, df and *R*^2^ values as follows: *t* = 0.13, df = 12 and *R*^2^ = 0.0014 for LI-II; *t* = 0.43, df = 10, and *R*^2^ = 0.0182 for LII-IV; *t* = 2.698, df = 20, and *R*^2^ = 0.2669 for LV-VI. *F* test values (F, DFn, and Dfd) were L I-II (42.44, 5, and 7), L III-IV (1.29, 5, and 5), and L V-V1 (1.96, 9, and 11).

Next, we determined the dendritic arborization and complexity by reconstructing Golgi-Cox staining neurons (Figure 4A). Sholl analysis showed a significant reduction in the number of branches at ∼40 μm in layers I–II and at ∼10–40 μm in layers V–VI in pups from diabetic rats compared with the control group (Figure 4B). However, only deep-layer neurons (V–VI) showed a significant decrease in DCI compared to control animals, suggesting significant functional consequences in neuron projection to extracortical areas (Figure 4C).

#### Reduced Excitability of Deep Cortical Neurons in the Offspring of Diabetic Rats

**FIGURE 5:**
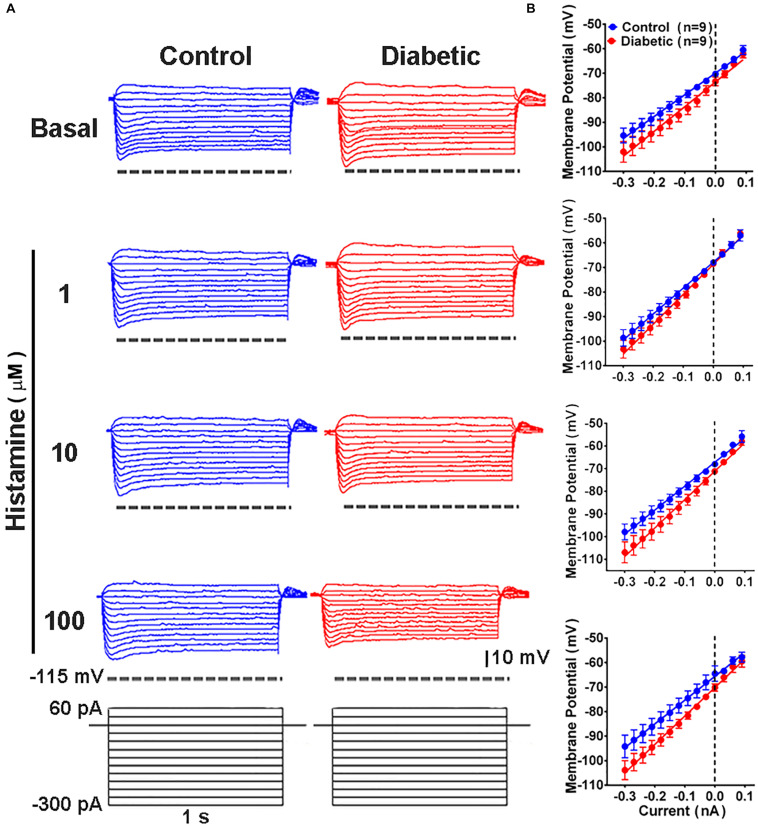
Current/voltage relationship in deep cortical primary motor cortex neurons of 21-days-old offspring from control and diabetic rats. **(A)** Representative traces of the changes in the membrane potential induced by a series of positive and negative current steps (1 s, 30 pA increments from –300 pA) from deep cortical layer neurons of control (blue) and diabetic (red) groups, in the absence (basal, upper traces) and presence of the indicated concentrations of histamine (lower traces). **(B)** Quantitative analysis. Values are means ± SEM from 9 neurons for each condition from 3 independent experiments. The line is the best-fit linear regression for the control (blue) and diabetic (red) groups. Values for passive electrophysiological properties are given in Table 1.

To determine whether DCI alterations resulted in changes in the electrophysiological membrane properties of deep-layer neurons, we examined the passive and active properties of layers V and VI pyramidal neurons in the absence and presence of histamine, which modulates neuronal excitability (Haas et al., 2008; Wu et al., 2019). Current/voltage curves (Figure 5) showed that in neurons from control animals, histamine increased in a concentration-dependent manner the resting membrane potential (RMP), input resistance (Rn), and sag conductance (Table 1). In contrast, in the offspring of diabetic rats we only observed an increase in RMP at 1 and 100 μM histamine (Table 1). However, the highest histamine concentration tested failed to increase the membrane potential to the level observed in the control group (Table 1). These results suggested changes in the potassium (K^+^) conductances of cortical neurons in animals from the diabetic group.

**TABLE 1:** Electrophysiological properties of deep cortical layer neurons in P21 offspring of control and diabetic rats.

		Control (*n* = 9)				Diabetic (*n* = 9)		
	Basal	Histamine (μM)			Basal	Histamine (μM)		
		1	10	100		1	10	100
RMP (mV)	−70.0 ± 1.2	−69.1 ± 0.7	−68.6 ± 0.7	−64.1 ± 0.8**	−74.6 ± 1.5	−69.0 ± 0.8*	−70.3 ± 1.9	−69.9 ± 1.3*^,a^
Rin (MΩ)	95.5 ± 11.8	122.9 ± 11.0*	126.6 ± 10.0**	144.6 ± 15.4***	132.0 ± 16.0	146.1 ± 18.5	151.6 ± 18.7	137.7 ± 20.5
τ (s)	15.5 ± 2.3	14.6 ± 32.7	17.8 ± 3.7	14.1 ± 3.0	11.5 ± 3.9	12.8 ± 3.4	13.1 ± 2.3	14.0 ± 2.6
Rheobase (pA)	172.2 ± 18.8	150.0 ± 14.4	138.8 ± 21.6	155.5 ± 37.6	255.5 ± 38.5	216.6 ± 42.4	244.4 ± 36.7	266.6 ± 42.4
Sag (mV)	3.83 ± 0.46	4.86 ± 0.38	5.37 ± 0.59*	5.68 ± 0.77**	3.78 ± 0.85	5.06 ± 0.78	4.18 ± 0.84	3.65 ± 0.56

Passive membrane properties were obtained from experiments illustrated in Figure 5. The membrane time constant (τ) was obtained at −30 pA and sag values at −300 pA. For differences between groups: ^*a*^P < 0.05 versus the control group, repeated measures two-way ANOVA and post hoc Sidak’s multiple comparison test. For differences in the same group: *P < 0.05, **P < 0.01, ***P < 0.001 versus basal values obtained from repeated-measures two-way ANOVA and post hoc Dunnett’s multiple comparisons test.

**FIGURE 6:**
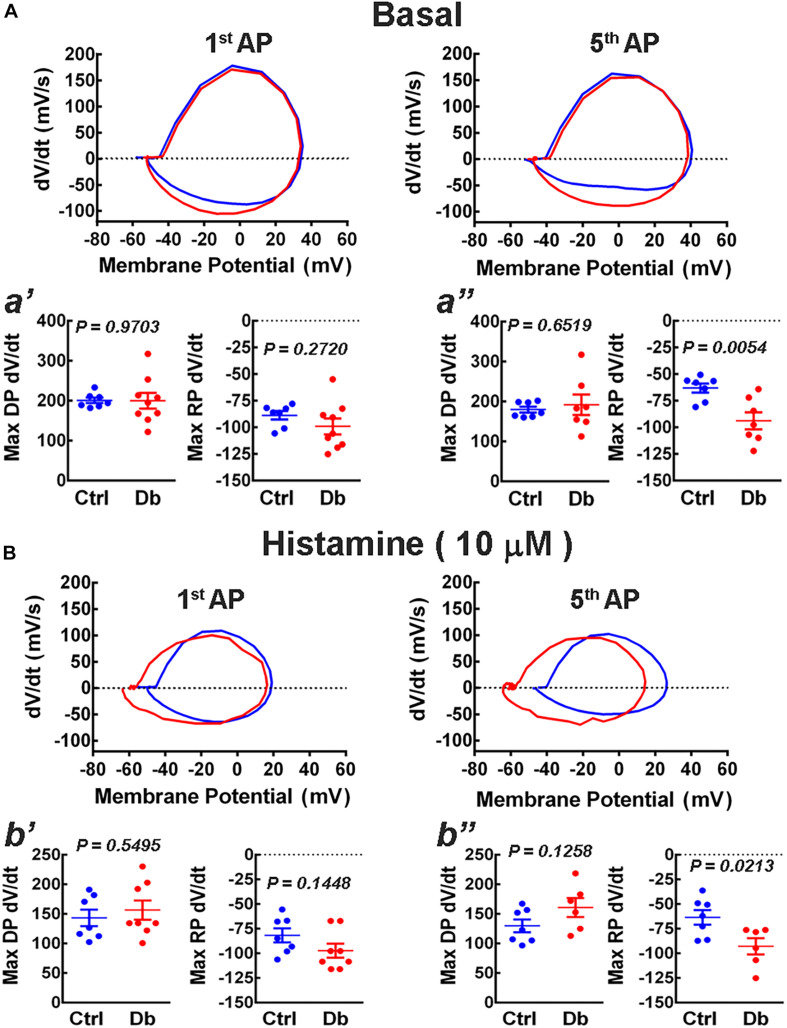
Phase plot analysis of action potentials. **(A,B)** Representative phase plots obtained from the first (1st AP) and fifth (5th AP) action potentials from the deep layers cortical neurons from the offspring of control (blue) and diabetic (red) rats in the absence (**A**, basal) or presence of 10 μM histamine **(B)**. Action potentials were induced by the injection of depolarizing current (300 pA) for 1 s. The analysis was performed on the maximum rate of the depolarization and repolarization phases of the 1st (**a′**; Max DP dV/dt: *t* = 0.03 df = 14, and *R*^2^ = 0.0001; *F* test values, *F* = 10.42, DFn = 8, and Dfd = 6 and Max RP dV/dt: *t* = 1.14, df = 14, and *R*^2^ = 0.0854; *F* test values, *F* = 4.50, DFn = 8, and Dfd = 6) and 5th AP under basal condition [**a′′**; Max DP dV/dt: *t* = 0.46 df = 12, and *R*^2^ = 0.0175; *F* test values, *F* = 12.79, DFn = 6, and Dfd = 6 and Max RP (dV/dt: *t* = 3.38 df = 12, and *R*^2^ = 0.4882; *F* test values, *F* = 3.48, DFn = 6, and Dfd = 6)] or histamine stimulation (**b′**: Max DP dV/dt: *t* = 0.61 df = 13, and *R*^2^ = 0.0282; *F* test values, *F* = 1.54, DFn = 7, and Dfd = 6 and Max RP dV/dt: *t* = 1.55, df = 13, and *R*^2^ = 0.1562; *F* test values, *F* = 1.12, DFn = 7, and Dfd = 6 and **b′′**; Max DP dV/dt: *t* = 1.65 df = 11, and *R*^2^ = 0.1997; *F* test values, *F* = 1.87, DFn = 5, and Dfd = 6 and Max RP dV/dt: *t* = 2.68, df = 11, and *R*^2^ = 0.3956; *F* test values, *F* = 1.04, DFn = 5, and Dfd = 6) for the Control (Ctrl) and Diabetic (Db) groups. Values are means ± SEM from 3 independent experiments. *P*-values were obtained by Student’s *t*-test.

To analyze changes in the ionic currents that underlie the depolarization (Na^+^) and repolarization (K^+^) phases of the action potential, we constructed phase plots from the first and fifth action potentials elicited by current injection (300 pA), in the absence and presence of histamine (10 μM; Figure 6). We found an increase in the maximum rate of the repolarizing phase, due to the activation of K^+^ conductances, for the 5th action potential in the diabetic group in both basal and stimulated conditions.

**FIGURE 7:**
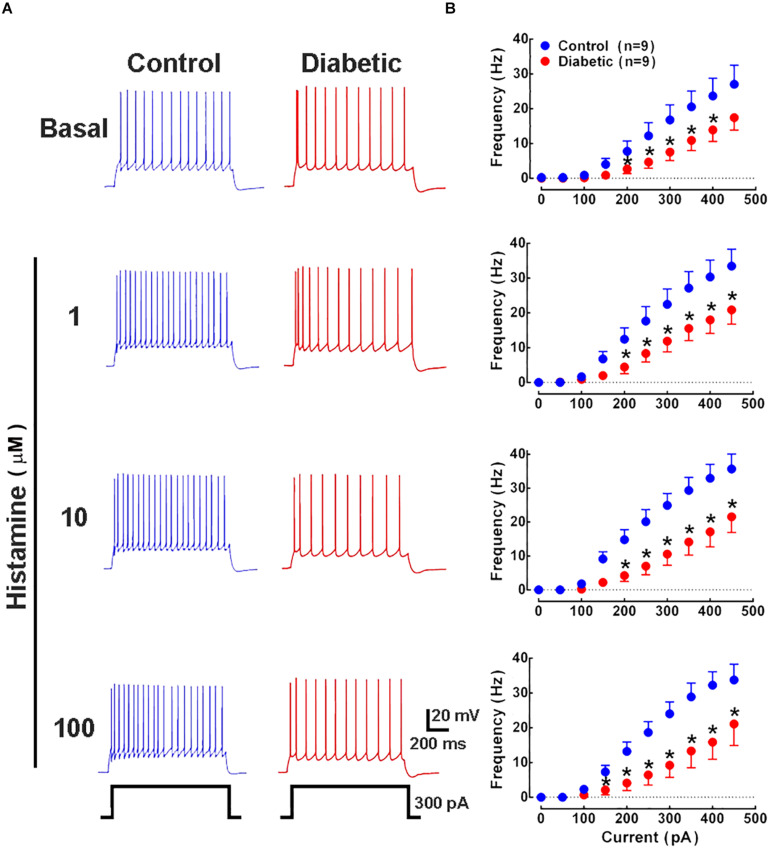
Analysis of action potential frequency in deep cortical neurons. **(A)** Representative traces of the action potentials generated by a depolarizing pulse (300 pA, 1 s) in the absence and presence of histamine (1, 10, and 100 μM) in deep cortical neurons of the offspring of control (blue) and diabetic (red) rats. **(B)** Current-frequency relationship. Values show the frequency (Hz) induced by depolarizing currents (0–500 pA) and are means ± SEM from 3 independent experiments. *P < 0.05 when compared with the control group, unpaired Student’s *t*-test.

Histamine increases neuronal excitability in several regions of the CNS such as the thalamus (McCormick and Williamson, 1991), layer 6b of the primary somatosensory cortex (Wenger Combremont et al., 2016), entorhinal cortex (Cilz and Lei, 2017), ventral pallidum (Ji et al., 2018), striatum (Zhuang et al., 2018), and spinal cord (Wu et al., 2019). Therefore, we recorded deep layer neurons from P21 pups by injecting depolarizing current in increasing steps under basal and stimulated (1–100 μM histamine) conditions. Our results showed a significant reduction in the action potential frequency from 200 pA in all experimental conditions in pups from diabetic rats compared with the control group (Figures 7A,B), without affecting the rheobase value (Table 1).

To evaluate if reduced H_1_R density underlies the altered response to histamine in the diabetic group, H_1_R and histamine levels were determined in the cerebral cortex of P21 pups. Binding assays showed no difference between groups in H_1_R density (50 ± 4 and 52 ± 9 fmol/mg of protein for the control and diabetic groups, respectively). Interestingly, a significantly higher amount of histamine was determined in the offspring of diabetic rats compared to the control group (354 ± 19 and 261 ± 35 ng/mg of protein, respectively; *P* = 0.0476, unpaired Student’s *t*-test).

## Discussion

In humans, maternal diabetes has been related to postnatal neurological impairment and psychiatric disorders due to fetal programming promoted by an inadequate environment (Ornoy et al., 1999, 2001; Bolaños et al., 2015; Márquez-Valadez et al., 2018), although these relationships are still poorly understood. Increased neurogenesis and early neuron maturation have been reported during corticogenesis in murine embryos exposed to high glucose (Fu et al., 2006; Solís et al., 2017). However, to our knowledge, the postnatal consequences on neocortical cytoarchitecture and function have not been reported. Therefore, in this study we explored changes in the cytoarchitecture, morphology, and neuronal function of the neocortex, focusing on M1 of neonates and P21 pups from diabetic rats.

At the cytoarchitectonic level, we found alterations in the distribution and expression of the transcription factors SATB2, FOXP2, and TBR1 at P0 and P21 that suggest dysregulation of cortical neurogenesis during embryo development associated with increased neurogenesis and impaired migration.

Increased neurogenesis is supported by higher SATB2, FOXP2, and TBR1 levels in M1, and by results previously reported for embryos of diabetic mice and rats (Fu et al., 2006; Solís et al., 2017). However, results obtained in this study for the neuronal markers SATB2 and TBR1 showed discrepancies in neonates of diabetic dams.

In the diabetic group, SATB2 showed a significant increase by immunofluorescence and Western blot, with no change in mRNA levels, which could be explained by post-transcriptional regulation and degradation of Satb2 mRNA through microRNAs. Although microRNAs have not been assessed in brain tissues from the offspring of diabetic mothers, increased expression of miR-15a and -15b has been reported in the skeletal muscle of adult offspring of diabetic women (Houshmand-Oeregaard et al., 2018), suggesting that increased microRNA levels may also occur in other tissues. The increase in TBR1 showed by immunofluorescence and qRT-PCR, with no change in the corresponding protein by Western blot, is more challenging to explain. However, one possibility is that heterogeneous changes in subareas of the frontal cortex may occlude detecting a net increase in protein.

An *ex vivo* study by Rash et al. (2018) reported impaired RG migratory scaffolding under high glucose environmental due to disruption of RG mitochondrial function or Ca^2+^ transport in the RG fibers. Cell migration is an essential mechanism for cortical lamination and function, and the protein Reelin is a crucial signal for neuronal migration, R.G. scaffolding, cell polarity establishment, and neuron maturation (Long et al., 2020). We found a decrease in Reelin immunoreactivity in the diabetic group (Figure 3B), suggesting that the stop signal provided by Reelin could be lost or at least reduced in the diabetic group during corticogenesis. This change may, in turn, lead to the expansion of the transcription factor domains of deep cortical markers to upper layers, as evidenced by the immunoreactivity pattern of SATB2, FOXP2, and TBR1 in the M1 of P21 offspring from diabetic rats. Moreover, impaired cell migration in the diabetic group is also suggested by the aberrant distribution of MAP2 (P0) and Nestin (E14), and the anomalous insertion of the apical dendrite into the neuron soma (P21).

The aberrant distribution of the transcription factors observed in the offspring of diabetic dams may have additional consequences for the establishment of neuronal networks. SATB2 regulates the development of cortico-thalamic and cortico-striatal pathways, as well as the formation of callosal projections in the superficial layers (Srinivasan et al., 2012; Sohur et al., 2014; Leone et al., 2015), and changes in the expression and distribution of this transcription factor suggest alterations in layer stratification and the establishment of neuronal pathways. Indeed, *Satb2*-deficient neurons fail to form the corpus callosum and project instead to subcortical areas (Alcamo et al., 2008). Furthermore, early SATB2 expression induces TBR1 expression, which reinforces cortico-thalamic identity, and Tbr1 mutants present callosal agenesis (Hevner et al., 2001), indicating that TBR1 is essential for the phenotype of callosal projection neurons. Hence, it is feasible that in the offspring of diabetic rats, the impaired distribution of callosal, subcortical, and cortico-thalamic projection neurons reported here promotes significant changes in forming these pathways with functional consequences, aspects relevant to explore in the future.

During corticogenesis, FOXP2 has been related to the acquisition and execution of motor sequences related to language in humans, singing in birds and ultrasonic vocalization in rodents (Groszer et al., 2008; Enard et al., 2009; Tsui et al., 2013; Vicario, 2013; Castellucci et al., 2016, 2017; Chabout et al., 2016; Chen et al., 2016). Impaired motor development and language performance have been related to maternal diabetes in school-age children (Ornoy et al., 1999; Ornoy et al., 2001; Bolaños et al., 2015), and to the best of our knowledge, this is the first study to explore the postnatal expression of this transcription factor in the offspring of diabetic dams. Here, we show increased expression and aberrant layer distribution of FOXP2 in the neocortex. Hence, higher and impaired ultrasonic vocalization and increased voluntary movement related to this task may also be expected. This possibility is supported by increased ultrasonic vocalization in pup mice that overexpress the human FOXP2 and by the severe motor impairment after maternal separation of pups with the disruption of both copies of the Foxp2 gene (Shu et al., 2005; Enard et al., 2009; Hammerschmidt et al., 2015). Furthermore, it has been recently reported that infants of mothers with diabetes show a higher incidence of attention deficit hyperactivity disorder (Lin et al., 2019).

Of note, FOXP2 regulates dopamine and cortico-striatal functions related to motor learning and working memory (Vargha-Khadem et al., 2005; Schulze et al., 2018). Heterozygous FOXP2 disruption reduces dendritic arbors and spines of striatal medium spiny neurons, decreases cortico-striatal long-term depression, and induces abnormal high firing rates during motor tasks in mice (Enard, 2011; French et al., 2012). Reelin is also critically involved in dendritic development (Niu et al., 2008; Chameau et al., 2009), and together these data suggest that an increase in FOXP2 is implicated in reduced dendritic complexity and excitability of pyramidal neurons, particularly of those of the deep neocortical layers, resulting in altered ultrasonic vocalization. In this study, Sholl and DCI analyses at P21 showed aberrant dendritic arborization, as well as Reelin reduction in neonates from diabetic rats. Together, previous reports and our results suggest that the increase in FOXP2 contributes to the decrease in dendritic complexity and excitability of pyramidal neurons, particularly of those of the deep neocortical layers.

Transcription factors are expected to be nuclear, however, post-transcriptional modifications, such as SUMOylation and phosphorylation, can affect nucleocytoplasmic transport. Here we observed a perinuclear/cytoplasmic localization of SATB2 and FOXP2 in control P0 offspring 2 h after birth, a phenomenon more evident in the diabetic group, suggesting that a fraction of these transcription factors may be inactive or have different gene targets in neonatal rats.

Both SATB2 and FOXP2 possess SUMOylation and phosphorylation consensus sequences (Szemes et al., 2006; Sheehan-Rooney et al., 2010; Goswami et al., 2012; Meredith et al., 2016; Blane et al., 2018). In particular, SUMOylation depends on the expression of Ubc9, and Watanabe et al. (2008) reported Ubc9 RNAm in the cortex of P0, P7, P14, P20, and 3-months-old rats, with the highest expression in P0 and the lowest in adult rats. Although the Ubc9 protein showed a similar pattern as the mRNA, the former was assessed at ages later than P7 (Watanabe et al., 2008).

Interestingly, SUMOylation is involved in SATB2 perinuclear localization (Dobreva et al., 2003), which suggests that higher SUMOylation may be occurring in P0 cortex of diabetic dams reported here. To study the expression of SUMO proteins, as well as that of AOS1/UBA2 (E1 heterodimer), Ubc9 (E2 enzyme), and PIAS proteins (E3 enzymes) in P0 offspring of control and diabetic rats would help to explain the perinuclear/cytoplasmic localization of SATB2 and FOXP2 observed here in P0 rats in control and high-glucose exposed embryos.

Phosphoproteomic analysis of neonatal murine brain (Goswami et al., 2012) also supports the possibility of changes in transcription factor activity in the neonatal brain. This study identified over 6,000 non-redundant phosphorylation sites in the neonatal murine brain compared to P21. Interestingly, two phosphorylation sites for SATB2 were found at P0 but not at P21. Changes in FOXP2 phosphorylation have also been proposed to participate in transcription regulation. For instance, Ser^557^ phosphorylation disrupts DNA binding due to electrostatic and steric hindrance (Blane et al., 2018).

Ubiquitination and phosphorylation are reversible, so the time after birth for tissue collection during P0 can also contribute to the differences observed in this study. However, this possibility is difficult to discuss since not all studies indicate the time after birth at which samples were obtained or due to differences in the day reported among studies.

We report here electrophysiological changes in deep cortical layer neurons in the P21 offspring of diabetic rats. Passive and active electrophysiological properties are tightly related to neuron morphology (Huang et al., 2017; Li et al., 2017; Papoutsi et al., 2017; Srinivas et al., 2017), and the diminished arborization near the soma and lower dendritic complexity reported in this study for the diabetic group suggest lower connectivity and neuron excitability disturbance.

Although there were no significant differences in the passive properties between groups under the basal condition, both the membrane input resistance (Rin) and time constant (τ) showed tendencies that match the change in DCI. The Rin showed a marked drift to increase compared to control cells, and the time constant is faster and requires less time to reach its charging value. Together, these modifications suggest a reduction in neuronal area that might be related to higher K^+^ conductance at resting membrane potential.

The lack of statistical significance may also be related to the age of the animals, and it is likely that these changes consolidate in adult animals. In this regard, the adult brain (≥90 days old) might present a more significant reduction in the dendritic arbor of pyramidal neurons with pronounced changes in passive/active electrophysiological membrane properties.

In contrast, the electrophysiological active properties were altered leading to lower excitability under basal and stimulated conditions, which may be explained by an increase in K^+^ conductances that contribute to repolarization in the cell soma, such as BK calcium-activated potassium channels, Kv4 family channels that mediate the A-type current (*I*_A_), and Kv3 family channels (Bean, 2007).

In several brain nuclei histamine increases neuronal excitability through a dual-action mediated by H_1_ and H_2_ receptors (see for instance McCormick and Williamson, 1991; Ji et al., 2018; Zhuang et al., 2018). The H_1_R-mediated excitatory effect involves a mixed ionic mechanism, namely closing of K^+^ channels, activation of Na^+^ channels and stimulation of the Na^+^-Ca^2+^ exchanger (McCormick and Williamson, 1991; Cilz and Lei, 2017; Wu et al., 2019). Information regarding cortical neurons is scarce, but Wenger Combremont et al. (2016) reported that histamine depolarizes and increases the firing frequency of slow-bursting neurons in mouse layer 6b of the primary somatosensory cortex. In rat entorhinal cortex via the combined activation of H_1_ and H_2_ receptors, the amine depolarizes a subset of GABAergic interneurons (Cilz and Lei, 2017).

Furthermore, the lower firing rate of cortical neurons observed here under basal and stimulated conditions in the diabetic group may be due to changes in their morphology. This notion is supported by a study reporting reduced excitability of postnatal cortical pyramidal neurons following altered neuronal migration, probably due to reduced afferent connectivity, suggesting that synaptic integration and circuit formation are impaired (Bocchi et al., 2017). Hence, reduced cortical afferents are expected in subcortical nuclei innervated by deep cortical neurons, such as thalamus and striatum, part of the complex network involved in learning and innate vocalization (Vargha-Khadem et al., 2005; Crosson, 2019; Mooney, 2020; Nieder and Mooney, 2020).

The increase in action potential frequency promoted by histamine in deep-layer cortical neurons is in accordance with the increased excitability previously reported for neurons in other regions of the CNS, namely thalamus (McCormick and Williamson, 1991), layer 6b of the primary somatosensory cortex (Wenger Combremont et al., 2016), entorhinal cortex (Cilz and Lei, 2017), ventral pallidum (Ji et al., 2018), striatum (Zhuang et al., 2018), and spinal cord (Wu et al., 2019). Furthermore, the lack of difference in H_1_R density supports that the electrophysiological changes observed in the offspring of diabetic rats rely mainly on morphological alterations. Of note, only deep-layer neocortical neurons were studied, and it will be important to examine in subsequent studies the effects on the electrophysiological properties of all cortical layers.

The consequences of the increased expression and changes in the laminar distribution of SATB2, TBR1, and FOXP2 are challenging to explain because most of the information previously reported relates to low or null expression in mutant animals. However, the abnormal distribution of the transcription factors and the electrophysiological alterations reported in this study suggest aberrant pathway formation and impaired cortical functions in the offspring of diabetic dams.

In summary, the expression and distribution of SATB2, TBR1, and FOXP2 suggest that high-glucose insult during development promotes complex changes in migration, neurogenesis, cell polarity establishment, and dendritic arborization, which in turn lead to reduced excitability of deep layers neurons. According to the role of the transcription factors investigated, our data suggest alterations in the cortico-cortical and extra-cortical innervation, a notion supported by the lower excitability of deep-layer neurons found. The postnatal effects of high glucose during fetal development *in vivo* are difficult to evaluate because of the low survival rate of pups. Nonetheless, it is essential to continue studying the postnatal effects in survivors, elucidate the mechanisms involved, and clarify the relationship between maternal diabetes and neurologic or psychiatric problems reported in humans. Furthermore, studies to evaluate other cortical functional areas, the integrity of extracortical pathways, and the functionality of other cortical layers and brain structures involved in voluntary movement must be performed to understand better the effect of maternal diabetes on offspring neurodevelopmental and fetal programming of pathologies such as autism, schizophrenia and attention-deficit hyperactivity syndrome, among others.

## Data Availability Statement

All datasets generated for this study are included in the article/Supplementary Material.

## Ethics Statement

The animal study was reviewed and approved by the Research and Animal Care (CICUAL), Biosecurity, and Ethics committees of Instituto Nacional de Perinatología Isidro Espinosa de los Reyes (Protocol number 3230-21202-01-2015).

## Author Contributions

All authors participated in results analysis and discussion and drafting of the manuscript. RV-B performed and analyzed most of the experiments. BM-V conducted and analyzed the E14 experiments. AF-C performed P21 immunofluorescence and processed data. GG-L completed image acquisition and processing. GH-L, EG, and EJG performed electrophysiology experiments and analysis. ND, J-AA-M, and AM-H participated in all steps of the study, including conception, critical revision of the manuscript, experimental analysis, and supervision. AM-H approved the final manuscript.

## Conflict of Interest

The authors declare that the research was conducted in the absence of any commercial or financial relationships that could be construed as a potential conflict of interest.

## Abbreviations

CNS: central nervous system
Ctip2: COUP-TF-interacting protein 2
Cux1: Cut like homeobox 1
E: embryo day
FoxP2: Forkhead box protein P2
M1: primary motor cortex
MAP2: microtubule-associated protein 2
MZ: marginal zone
NSCs: neural stem cells
RG: radial glia
Satb2: special AT-rich sequence-binding protein 2
Tbr1: T-box brain 1
VZ: ventricular zone.
