# Supplementary material for: Impaired Cortical Cytoarchitecture and Reduced Excitability of Deep-Layer Neurons in the Offspring of Diabetic Rats
Source: Front Cell Dev Biol. 2020 Sep 16;8:564561. doi: 10.3389/fcell.2020.564561 (PMC7527606; doi:10.3389/fcell.2020.564561)
Supplement: Supplementary file 1 [file Data_Sheet_1.docx]

***Supplementary Tables***

**Supplementary Table 1. Values of plasma glucose, and brain and body weight of newborn pups.**

| Group | n | Glucose  ( mg/dl ) | Brain weight  ( mg ) | Body weight  ( g ) |
| --- | --- | --- | --- | --- |
| Control | 20 | 89 ± 4 | 260 ± 4 | 6.6 ± 0.1 |
| Diabetic | 18 | 236 ± 18 **** | 217 ± 4 **** | 5.2 ± 0.1 **** |

Data are means ± SEM. *****P* < 0.0001, unpaired Student´s *t*-test.

**Supplementary Table 2. Birth and mortality index of the offspring per condition.**

| Group | Pregnant rats | Pups per  offspring | Live pups  (total) | Dead pups  (total) |
| --- | --- | --- | --- | --- |
| Control | 7 | 12.1 ± 1.5 | 12.0 ± 1.5 | 1.4 ± 0.14 |
| Diabetic | 7 | 11.3 ± 2.0 | 7.4 ± 2.2 | 3.9 ± 0.7**** |

Data are means ± SEM. *****P* < 0.0001, unpaired Student´s *t*-test.
