# Supplementary material for: Impaired Cortical Cytoarchitecture and Reduced Excitability of Deep-Layer Neurons in the Offspring of Diabetic Rats
Source: Front Cell Dev Biol. 2020 Sep 16;8:564561. doi: 10.3389/fcell.2020.564561 (PMC7527606; doi:10.3389/fcell.2020.564561)
Supplement: Supplementary file 2 [file Data_Sheet_2.pdf]

Supplementary Figures:

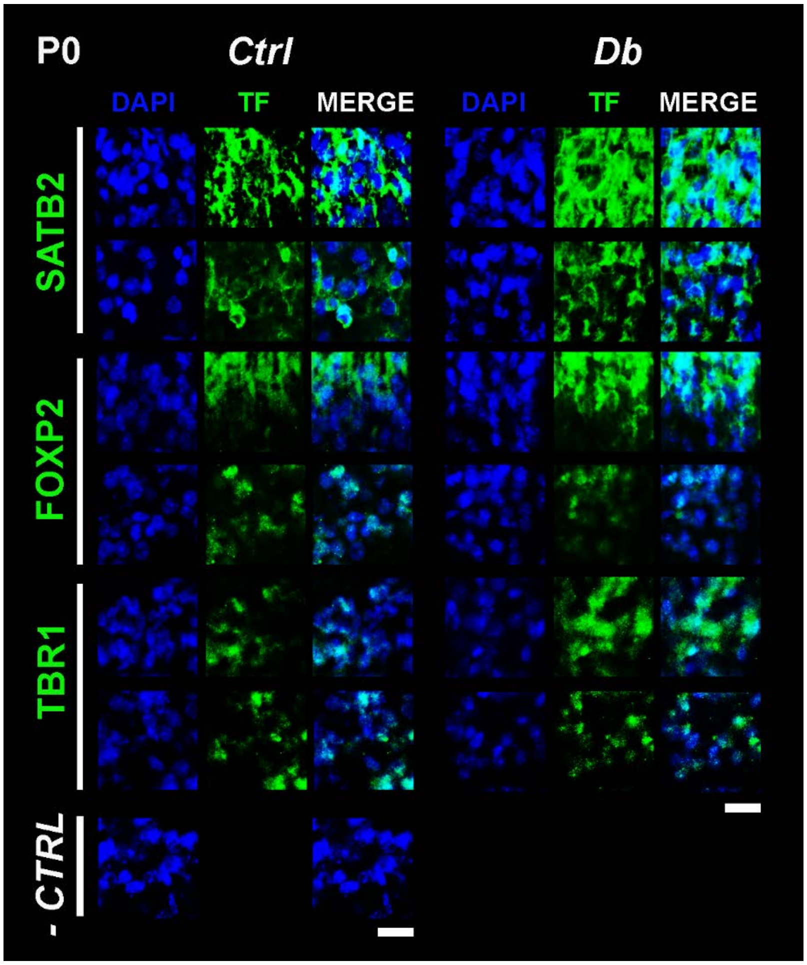

Supplementary Figure 1. SATB2, FOXP2, and TBR1 immunofluorescence in the neonatal primary motor cortex from control and diabetic dams. Representative micrographs (40×) of DAPI-stained nuclei (blue) and SATB2, FOXP2, and TBR1 (green) immunodetection, at postnatal day zero (P0) from Control (Ctl) and Diabetic (Db) groups (n = 3-4) taken from upper (U) and deeper layers. Scale bars = 25 µm. -Ctl = negative control.

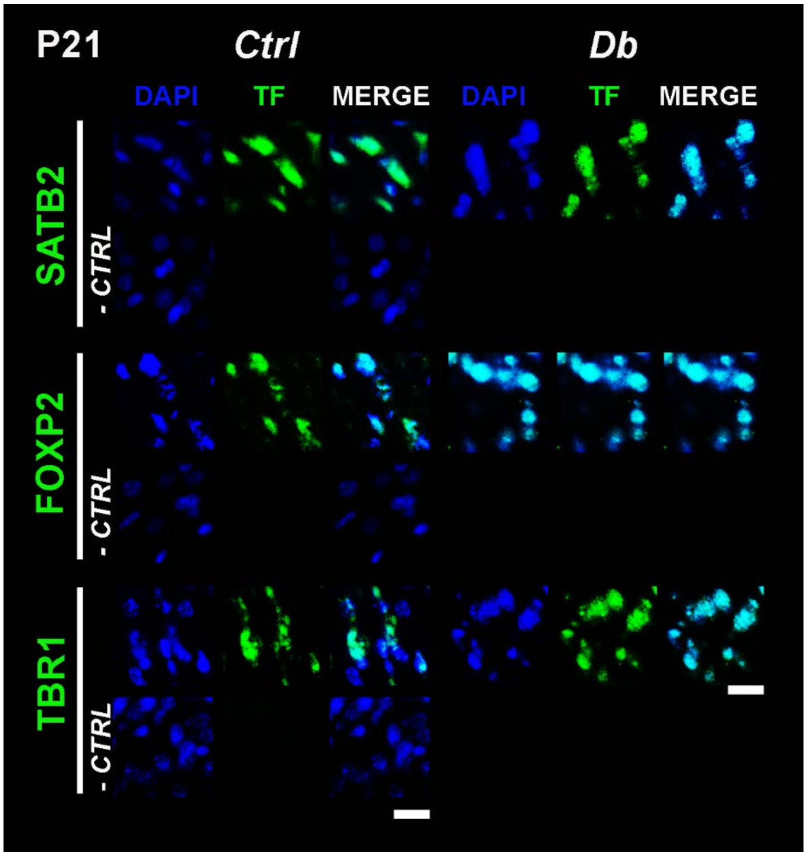

Supplementary Figure 2. SATB2, FOXP2, and TBR1 distribution in the primary motor cortex of 21-day old offspring from control and diabetic rats. Representative micrographs (40×) of DAPI-stained nuclei (blue) and SATB2, FOXP2, and TBR1 (green) immunodetection, at postnatal day 21 (P21) from Control (Ctl) and Diabetic (Db) groups (n = 3-4). Scale bars = 25 µm. -Ctl = negative control.
